# Supplementary material for: Filtering Eye-Tracking Data From an EyeLink 1000: Comparing Heuristic, Savitzky-Golay, IIR and FIR Digital Filters
Source: J Eye Mov Res. 2023 Oct 19;14(3):10.16910/jemr.14.3.6. doi: 10.16910/jemr.14.3.6 (PMC11219126; doi:10.16910/jemr.14.3.6)
Supplement: Supplementary file 1 [file jemr-14-03-f-SD1-01.pdf]

Appendix

EyeLink from SR research

Here are the instructions for how to turn off the heuristic filters for the EyeLink 1000 and 1000 plus.

- Run the EyeLink Software on the Host PC.
- Click on **Set Options** on the **Camera Setup** page.
- **File Sample filter** option is now visible.
- Turn it **OFF** for collecting data Unfiltered, **STD** for standard filtering and **EXTRA** for extra filtering.

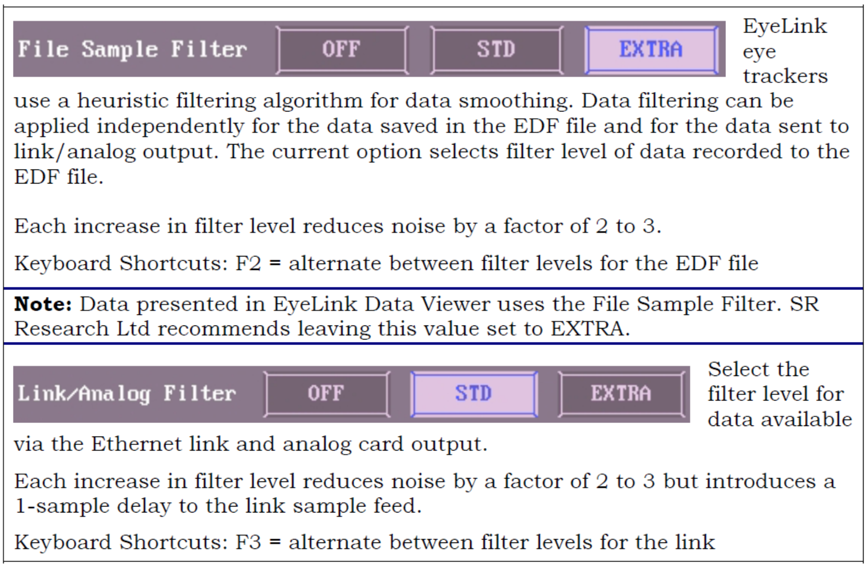

File Sample Filter | OFF | STD | EXTRA

EyeLink eye trackers use a heuristic filtering algorithm for data smoothing. Data filtering can be applied independently for the data saved in the EDF file and for the data sent to link/analog output. The current option selects filter level of data recorded to the EDF file.

Each increase in filter level reduces noise by a factor of 2 to 3.

Keyboard Shortcuts: F2 = alternate between filter levels for the EDF file

Note: Data presented in EyeLink Data Viewer uses the File Sample Filter. SR Research Ltd recommends leaving this value set to EXTRA.

Link/Analog Filter | OFF | STD | EXTRA

Select the filter level for data available via the Ethernet link and analog card output.

Each increase in filter level reduces noise by a factor of 2 to 3 but introduces a 1-sample delay to the link sample feed.

Keyboard Shortcuts: F3 = alternate between filter levels for the link

Appendix Figure 1. Filtering options of EyeLink 1000 plus. Screenshot from EyeLink 1000 Plus User Manual version 1.0.12 (Page 35).

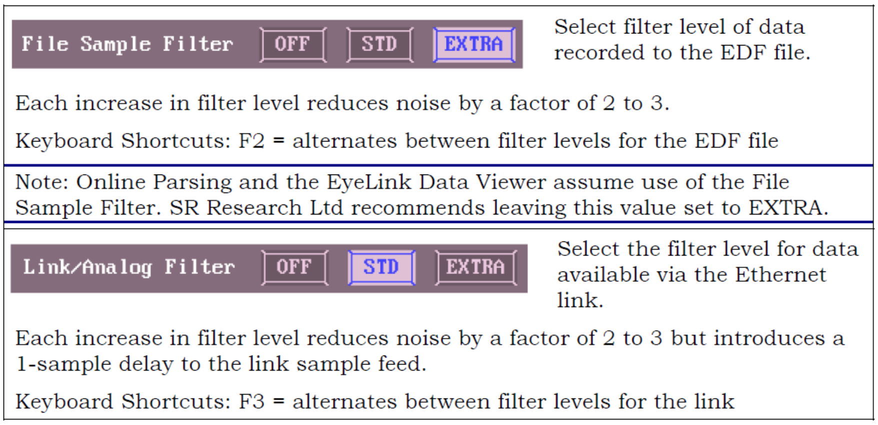

File Sample Filter | OFF | STD | EXTRA

Select filter level of data recorded to the EDF file.

Each increase in filter level reduces noise by a factor of 2 to 3.

Keyboard Shortcuts: F2 = alternates between filter levels for the EDF file

Note: Online Parsing and the EyeLink Data Viewer assume use of the File Sample Filter. SR Research Ltd recommends leaving this value set to EXTRA.

Link/Analog Filter | OFF | STD | EXTRA

Select the filter level for data available via the Ethernet link.

Each increase in filter level reduces noise by a factor of 2 to 3 but introduces a 1-sample delay to the link sample feed.

Keyboard Shortcuts: F3 = alternates between filter levels for the link

Appendix Figure 2. Filtering options of EyeLink 1000. Screenshot from EyeLink 1000 User Manual version 1.5.0 (Page 18).

iViewX from SMI

Here are the instructions for how to use the heuristic filters for the iViewX from SMI.

- Click on **Setup**.
- **Eye Data Filtering** option is now visible. See the following screenshot for assistance.

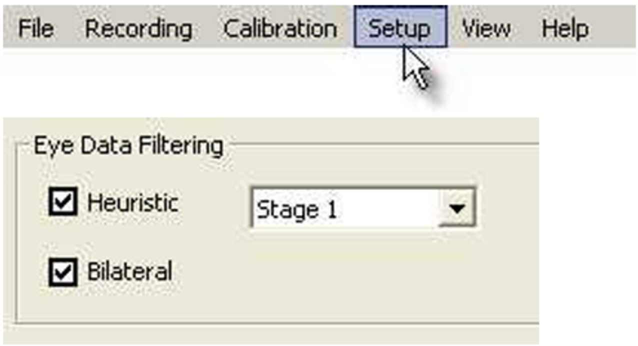

The *Input Filter Eye* filters all incoming eye data. Two different filters can be cascaded:

Heuristic Filter: Removes noise by cleaning the data from peaks.
Stage 1 removes impulse noise of 1 sample. A latency of 1 sample is added to the data.
Stage 2 removes ramp noise of 2 samples. A latency of 2 samples is added to the data.

Appendix Figure 3. Filtering options of IViweX. Screenshot from IViewX User Manual version 2.8 (Page 339)

These instructions are taken from the User Manual available at

EyeLink 1000 plus (version 1.0.12) – https://risoms.github.io/mdl/docs/build/manual/Eye-Link%201000%20Plus%20User%20Manual%201.0.12.pdf

EyeLink 1000 (version 1.5.0) - http://sr-research.jp/support/EyeLink%201000%20User%20Man-ual%201.5.0.pdf

IViewX (version 2.8) - https://tsgdoc.socsci.ru.nl/images/6/6f/IViewX.pdf
